# Outcome of acute respiratory distress syndrome in university and non-university hospitals in Germany

**DOI:** 10.1186/s13054-017-1687-0

**Published:** 2017-05-30

**Authors:** Konstantinos Raymondos, Tamme Dirks, Michael Quintel, Ulrich Molitoris, Jörg Ahrens, Thorben Dieck, Kai Johanning, Dietrich Henzler, Rolf Rossaint, Christian Putensen, Hermann Wrigge, Ralph Wittich, Maximilian Ragaller, Thomas Bein, Martin Beiderlinden, Maxi Sanmann, Christian Rabe, Jörn Schlechtweg, Monika Holler, Fernando Frutos-Vivar, Andres Esteban, Hartmut Hecker, Simone Rosseau, Vera von Dossow, Claudia Spies, Tobias Welte, Siegfried Piepenbrock, Steffen Weber-Carstens

**Affiliations:** 1 Department of Anaesthesiology and Intensive Care Medicine, Hannover Medical School, Carl-Neuberg-Straße 1, 30625 Hannover, Germany; 2 Department of Cardiology, KRH Klinikum Robert Koch Gehrden, Gehrden, Germany; 3 Department of Anaesthesiology, Emergency and Intensive Care Medicine, Göttingen University Hospital, Göttingen, Germany; 4 Department of Cardiothoracic, Transplantation and Vascular Surgery, Hannover Medical School, Hannover, Germany; 5 Department of Anaesthesiology and Intensive Care Medicine, Klinikum Links der Weser, Bremen, Germany; 6 Department of Anaesthesiology, Herford Hospital, Herford, Germany; 7 Department of Anaesthesiology, RWTH Aachen University Hospital, Aachen, Germany; 8 Department of Anaesthesiology and Surgical Intensive Care Medicine, Bonn University Hospital, Bonn, Germany; 9 Department of Anaesthesiology and Intensive Care Medicine, Leipzig University Hospital, Leipzig, Germany; 10 Department of Anaesthesiology and Intensive Care Medicine, Carl Thieme Hospital, Cottbus, Germany; 11 Department of Anaesthesiology and Intensive Care Medicine, Carl Gustav Carus University Hospital, Dresden, Germany; 12 Department of Anaesthesiology, Regensburg University Hospital, Regensburg, Germany; 13 Department of Anaesthesiology and Intensive Care Medicine, Essen University Hospital, Essen, Germany; 14 Department of Anaesthesiology, Dietrich-Bonhoeffer Hospital, Neubrandenburg, Germany; 15 Department of Internal Medicine, Bonn University Hospital, Bonn, Germany; 16 Department of Anaesthesiology, Klinikum Bad Salzungen, Bad Salzungen, Germany; 17 Department of Anaesthesiology and Intensive Care Medicine, Municipal Hospital Martha-Maria Halle-Dölau, Halle, Germany; 18 Department of Intensive Care Unit, Hospital Universitario de Getafe, CIBER de Enfermedades Respiratorias, Madrid, Spain; 19 Department of Biometry, Hannover Medical School, Hannover, Germany; 20 Department of Internal Medicine, Division Infectiology and Pulmonology, Charité University Hospital, Berlin, Germany; 21 Department of Anesthesiology and Intensive Care, Ludwig-Maximilians-Universität München, Geschwister-Scholl-Platz 1, 80539 München, Germany; 22 Department of Anaesthesiology and Intensive Care Medicine, Charité University Hospital, Berlin, Germany; 23 Department of Respiratory Medicine, Hannover Medical School, Hannover, Germany

**Keywords:** Acute respiratory distress syndrome, Care setting, Mechanical ventilation, Driving pressure, Biphasic positive airway pressure

## Abstract

**Background:**

This study investigates differences in treatment and outcome of ventilated patients with acute respiratory distress syndrome (ARDS) between university and non-university hospitals in Germany.

**Methods:**

This subanalysis of a prospective, observational cohort study was performed to identify independent risk factors for mortality by examining: baseline factors, ventilator settings (e.g., driving pressure), complications, and care settings—for example, case volume of ventilated patients, size/type of intensive care unit (ICU), and type of hospital (university/non-university hospital). To control for potentially confounding factors at ARDS onset and to verify differences in mortality, ARDS patients in university vs non-university hospitals were compared using additional multivariable analysis.

**Results:**

Of the 7540 patients admitted to 95 ICUs from 18 university and 62 non-university hospitals in May 2004, 1028 received mechanical ventilation and 198 developed ARDS. Although the characteristics of ARDS patients were very similar, hospital mortality was considerably lower in university compared with non-university hospitals (39.3% vs 57.5%; *p* = 0.012). Treatment in non-university hospitals was independently associated with increased mortality (OR (95% CI): 2.89 (1.31–6.38); *p* = 0.008). This was confirmed by additional independent comparisons between the two patient groups when controlling for confounding factors at ARDS onset. Higher driving pressures (OR 1.10; 1 cmH_2_O increments) were also independently associated with higher mortality. Compared with non-university hospitals, higher positive end-expiratory pressure (PEEP) (mean ± SD: 11.7 ± 4.7 vs 9.7 ± 3.7 cmH_2_O; *p* = 0.005) and lower driving pressures (15.1 ± 4.4 vs 17.0 ± 5.0 cmH_2_O; *p* = 0.02) were applied during therapeutic ventilation in university hospitals, and ventilation lasted twice as long (median (IQR): 16 (9–29) vs 8 (3–16) days; *p* < 0.001).

**Conclusions:**

Mortality risk of ARDS patients was considerably higher in non-university compared with university hospitals. Differences in ventilatory care between hospitals might explain this finding and may at least partially imply regionalization of care and the export of ventilatory strategies to non-university hospitals.

**Electronic supplementary material:**

The online version of this article (doi:10.1186/s13054-017-1687-0) contains supplementary material, which is available to authorized users.

## Background

Germany has the highest number of intensive care unit (ICU) beds and admissions per capita, and this has been related to the very low mortality due to sepsis compared with other countries [1]. Nevertheless, with the exception of one study performed in 1991 in Berlin [2] and studies including selected acute respiratory distress syndrome (ARDS) patients treated in referral centers [3–5], the characteristics and outcome of unselected, ventilated patients in Germany remain unknown.

The Second International Study of Mechanical Ventilation (Second VENTILA study) was carried out in 23 countries, and one in five of the included patients originated from Germany. However, because Germany did not participate in the First VENTILA study, patients from Germany were excluded from the comparative analyses [6, 7]. Therefore, in 2004 when a hospital mortality of 63.2% was observed in patients with ARDS [7], patients from Germany had not been included. Mortality in routine medical care, as reflected (at least partially) by observational cohort studies [6–13], is usually higher than in randomized trials [5, 14–20] in which, generally speaking, less severely ill patients are enrolled. Randomized trials [5, 14–20] are mainly conducted in university hospitals, as indeed are most observational studies [9–13]; however, these university centers represent only a very small proportion of all hospitals in which ARDS patients are treated.

Many non-university hospitals participated in the Second VENTILA study, in which the hospital mortality of ARDS patients was 15.4% higher than that found in a Spanish study conducted mainly in university hospitals (Acute Lung Injury: Epidemiology and Natural History (ALIEN) study) [7, 11]. Non-university hospitals also participated in the King County Lung Injury Project (KCLIP study), in which the low hospital mortality of 41.1% was partially related to local healthcare settings with early discharge to other hospitals, or to skilled nursing/rehabilitation facilities with acute care beds [1, 21]. In this KCLIP study, mortality in the non-university hospitals was 11.5% higher but patients were on average 17 years older and had higher illness severity scores compared with those in university hospitals [21]; whether the setting independently affects the outcome of ARDS remains unknown [9, 12, 22–24].

Many factors influence ARDS outcome [2–26] including the underlying disease, appropriate treatment, and the use of protective ventilation [15, 16, 24, 26]. Different types of hospitals may implement lung protective strategies to varying degrees. Also, taking into account other variables that different hospitals may introduce (e.g., case volume or staffing), we hypothesized that the mortality risk for ARDS might be increased in non-university hospitals compared with university hospitals, irrespective of patient characteristics or other factors. Therefore, this study aimed to determine whether different types of care settings have an impact on the survival of ARDS, and to assess differences in ventilatory care that might be related to ARDS outcome.

## Methods

As part of the Second VENTILA study [7], during a 1-month period we enrolled adult patients who were ventilated for at least 12 h invasively or 1 h non-invasively after admission to an ICU in Germany. All participating investigators and hospitals are listed in the Appendix. This study was approved by the ethical committee of the Medical School Hanover for all centers involved, and informed consent was waived (No. 3575).

ARDS was defined according to the American–European Consensus Conference; patients with acute lung injury (ALI) who did not progress to ARDS were not included in the analysis of the group diagnosed with ARDS [25]. On ICU admission, severity of illness was evaluated with the Simplified Acute Physiology Score II (SAPS II) and organ dysfunctions were evaluated daily as described by Esteban et al. [6–8].

ARDS patients treated in university hospitals were compared with those treated in non-university hospitals. The main outcome was overall hospital mortality: the variables of ventilatory care, as well as the incidence and incidence rates of complications, were analyzed to explain potential differences in mortality. Therapeutic ventilation was identified to exclude days of weaning by selecting only those ventilator days with FiO_2_ > 0.4 and PEEP > 5 cmH_2_O, which represented the minimum values as criteria for the start of weaning in the low tidal volume trial of the ARDS Network [16]. Depending on the distribution of data, Student’s *t* test or the Mann–Whitney *U* test was used to compare continuous data, and the chi-square test or Fisher’s exact test to compare proportions. The survival rate of ARDS patients was analyzed using the Kaplan–Meier method and the log-rank test was used to compare groups. *p* < 0.05 was considered statistically significant.

### Multivariable analyses

As described in detail in Additional file 1, factors independently associated with increased hospital mortality were determined using explorative multivariable logistic regression in which two multivariable models for patients with and without ARDS were employed. Briefly, we first selected potential prognostic factors by means of univariable analyses when variables were associated with hospital mortality with *p* < 0.15 and, in addition, also by entering predefined variables of potential clinical importance following an expert-based selection process. Because entering too many predictors into a model and collinearity between predictors can lead to unstable coefficient estimation, we then grouped these selected variables into four variable categories:

(i) Patients’ baseline factors: age, sex, SAPS II, main problem (medical/surgical), main reason for initiation of ventilation (pneumonia, lung disease other than pneumonia, sepsis, postoperative acute respiratory failure (ARF), other ARF, neurological reason), length of hospital stay prior to ventilation, and (only in the ARDS group) origin of ARDS (extrapulmonary/pulmonary).
(ii) Factors related to individual patient management: driving pressure, tidal volume per kilogram of predicted body weight, respiratory rate, plateau pressure, PEEP, FiO_2_, PaO_2_/FiO_2_, pH, dynamic compliance (mean of all ventilatory and gas exchange variables from the first week after onset of ARDS), successful non-invasive ventilation, high plateau pressure (>30 cmH_2_O) on 2 consecutive days, and use of sedatives or vasoactive drugs on 2 consecutive days.
(iii) Complications during ventilation: metabolic acidosis (pH < 7.3 and PaCO_2_ < 45 mmHg), respiratory acidosis (pH < 7.35 and PaCO_2_ > 55 mmHg), pneumonia, barotrauma, hepatic failure, renal failure, shock, coagulopathy, and lowest PaO_2_/FiO_2_ ratio.
(iv) Factors related to the setting of care: size of hospital, size of the ICU, type of ICU (surgical/medical, medical, surgical, neurological), case volume of all patients admitted to the ICU, case volume of mechanically ventilated patients as well as case volume of mechanically ventilated patients per bed, and university/non-university hospital.

In Germany, a university hospital is organized by a federal state and is directly affiliated to a medical faculty with at least 150 new medical students per year and at least 60 professors teaching human medicine. Medical faculty and university hospitals are institutionally linked and cooperate within a joint governance structure. Research and teaching is institutionally ensured by a legal mandate with corresponding, independent funding. The hospital provides the infrastructure for all or most of the medical specialties that are necessary for teaching and research.

To select the variables for the final multivariable models we then performed multivariable analyses within each of these four variable categories separately for patients with and without ARDS. Only those variables contributing with *p* < 0.10 to the multivariable analysis per variable category were entered into the two overall final models, thereby correcting for collinearity of predictors.

In the group of patients with ARDS, the variables that contributed with *p* < 0.10 and were subsequently entered into the final model for multivariable logistic regression were: sex, SAPS II, driving pressure, FiO_2_, pH (mean values from the first week after onset of ARDS), lowest PaO_2_/FiO_2_, use of vasoactive drugs on 2 consecutive days, metabolic acidosis, hepatic failure, renal failure, and university/non-university hospital. (Building of the multivariable model for patients without ARDS is described in Additional file 1).

Finally, factors independently associated with hospital mortality were determined using a stepwise backward elimination procedure with a threshold of *p* < 0.05 according to Wald statistics. The goodness-of-fit of the final models was assessed using the Hosmer–Lemeshow test, and the discrimination ability was analyzed with reference to the receiver-operator characteristic (ROC), and its area under the curve (AUC) was calculated for the two final models for patients with and without ARDS.

### Control for confounding to verify differences in mortality risk in ARDS patients

Additional to and separate from the analyses of independent risk factors, a potential difference in mortality between ARDS patients in university and non-university hospitals was confirmed by an additional confirmatory analysis. This independent examination controlled for potential predefined confounding factors present at the onset of ARDS (e.g., demographics, comorbidity, severity of lung injury) by means of multivariable logistic regression without further variable selection, thereby minimizing selection bias due to potential structural differences between the two groups as well as avoiding overadjustment for events occurring later during ventilation when searching for causality.

Predefined potential confounders were derived from the literature [6, 22, 23] and the authors’ own analysis when factors were associated with the occurrence of ARDS (as presented in Table 1). Selection of those potential confounders eligible for statistical control was clearly defined a priori by means of clinical assessment, and not by statistical testing. The relevance of the differences between groups was only neglected when fulfilling the following predefined conditions: age < 2 years, SAPS II < 3 points, lung compliance < 3 cmH_2_O, PaO_2_/FiO_2_ < 10 mmHg, or differences < 2% for categorical variables. Nevertheless, we included factors when the differences were low but the standard deviations or interquartile ranges differed considerably.

**Table 1:** Demographic and clinical characteristics at onset of ARDS in university and non-university hospitals

	University hospital	Non-university hospital	
	(*n* = 87)	(*n* = 111)	*p* value
Age (years)	59.0 (16.2); 63 (47–72)	62.8 (15.9); 65 (52–74)	0.10
Sex, male/female	62 (71.3)/25 (28.7)	80 (72.1)/31 (27.9)	0.9
BMI (kg/m^2^)	26.0 (4.9); 25.7 (23.2–27.7)	26.9 (6.2); 26.0 (23.1–29.4)	0.34
BMI categories			
< 18.5/<25.5/<30.0/≥30.0 kg/m^2^	3 (4.5)/28 (41.8)/28 (41.8)/8 (11.9)	5 (4.7)/39 (36.8)/41 (38.7)/21(19.8)	0.6
SAPS II at ICU admission	45.2 (17.2); 42 (32–58)	48.7 (20.4); 48 (37–62)	0.21
Main problem, medical/surgical	48 (55.2)/39 (44.8)	69 (62.2)/42 (37.8)	0.32
ARDS origin, pulmonary/ extrapulmonary	65 (78.3)/18 (21.7)	79 (72.5)/30 (27.5)	0.36
Late onset of ARDS^a^	35 (40.2)	28 (25.2)	0.02
Onset of ARDS: day after initiation of mechanical ventilation	1 (0-5)	0 (0-2)	0.02
PaO_2_/FiO_2_ (mmHg)	157 (124-186)	149 (114-179)	0.29
Dynamic compliance^b^ (ml/mbar; ml/mbar/kg PBW)	39 (29–55); 0.58 (0.44–0.79)	33 (24–43); 0.52 (0.39–0.67)	0.006; 0.04
Reason for initiation of mechanical ventilation (only presented when different between groups)^c^			
Postoperative acute respiratory failure	19 (21.8)	12 (10.8)	0.03
Acute respiratory failure after aspiration	2 (2.3)	12 (10.8)	0.02
Complications until the onset of ARDS			
Sepsis^c^	41 (47.1)	38 (34.2)	0.07
Pneumonia^c^	43 (49.4)	44 (39.6)	0.17
Cardiovascular failure	63 (72.4)	71 (64.0)	0.21
Renal failure	26 (29.9)	32 (28.8)	0.87
Coagulopathy	23 (26.4)	8 (7.2)	<0.001
Liver failure	11 (12.6)	2 (1.8)	0.002
Metabolic acidosis	8 (9.2)	13 (11.7)	0.57
Respiratory acidosis	18 (20.7)	23 (20.7)	1.0
Barotrauma	14 (16.1)	13 (11.7)	0.37

Data presented as mean (standard deviation); median (interquartile range) or *n* (%)

*ARDS* acute respiratory distress syndrome, *BMI* body mass index, *SAPS II* Severe Acute Physiology Score II, *ICU* intensive care unit, *PaO*_*2*_ */FiO*_*2*_ arterial-to-inspired oxygen ratio, *PBW* predicted body weight, *PEEP* positive end-expiratory pressure

^a^Defined as ARDS developing ≥48 h after the onset of mechanical ventilation

^b^Compliance = tidal volume / (plateau pressure – PEEP)

^c^All cases as reason for initiation of mechanical ventilation or developing as complication during mechanical ventilation until the onset of ARDS. The other reasons for the initiation of mechanical ventilation did not differ between ARDS patients in university and non-university hospitals (e.g., multiple trauma, eight vs five patients (9.2 vs 4.5%), *p* = 0.19). Further details for these other reasons for the entire group of patients with ARDS are presented in Additional file 1: Table S1

Based on these criteria, differences between the groups in, for example, gender, PaO_2_/FiO_2_ at onset of ARDS, and prevalence of renal failure or respiratory acidosis at onset of ARDS were considered too small to distort the relationship with mortality and were not controlled for in the multivariable regression. In contrast, age, body mass index, SAPS II, main problem (medical/surgical), origin of ARDS (pulmonary/extrapulmonary), day of onset of ARDS, dynamic compliance at onset of ARDS, reasons for initiation of mechanical ventilation (postoperative ARF, ARF after aspiration, ARF after multiple trauma), and comorbidities or complications present until onset of ARDS (sepsis, pneumonia, cardiovascular failure, coagulation failure, liver failure, metabolic acidosis) were selected for statistical control of confounding.

To control for confounding, we performed a one-step multivariable logistic regression with hospital mortality as the outcome variable, and with the academic status of the hospital (university/non-university) and the potential confounders as covariates. The odds ratio (OR) was calculated as an effect measure for the mortality risk of ARDS patients in non-university hospitals compared with university hospitals. We added a sensitivity analysis to study the robustness of this multivariable model, varying the potential confounders by excluding factors or combinations of factors with missing values.

## Results

During May 2004, 7540 patients were admitted to the 95 participating ICUs from 80 hospitals in 64 German cities. Of these, 1028 were ventilated for ≥ 12 h (13.6%; 95% CI 12.9–14.4%): 914 patients were ventilated invasively and 114 non-invasively (of whom 56% were intubated). The hospital mortality rate of these 1028 ventilated patients was 30.1% (95% CI 27–33%) (Additional file 1: Table S1).

**Fig. 1:**
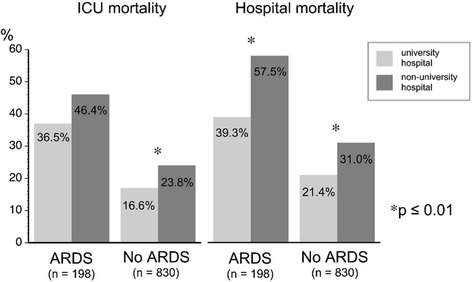
Mortality of mechanically ventilated patients during their stay in the intensive care unit (*ICU*) and hospital, in university and non-university hospitals. *ARDS* acute respiratory distress syndrome

**Fig. 2:**
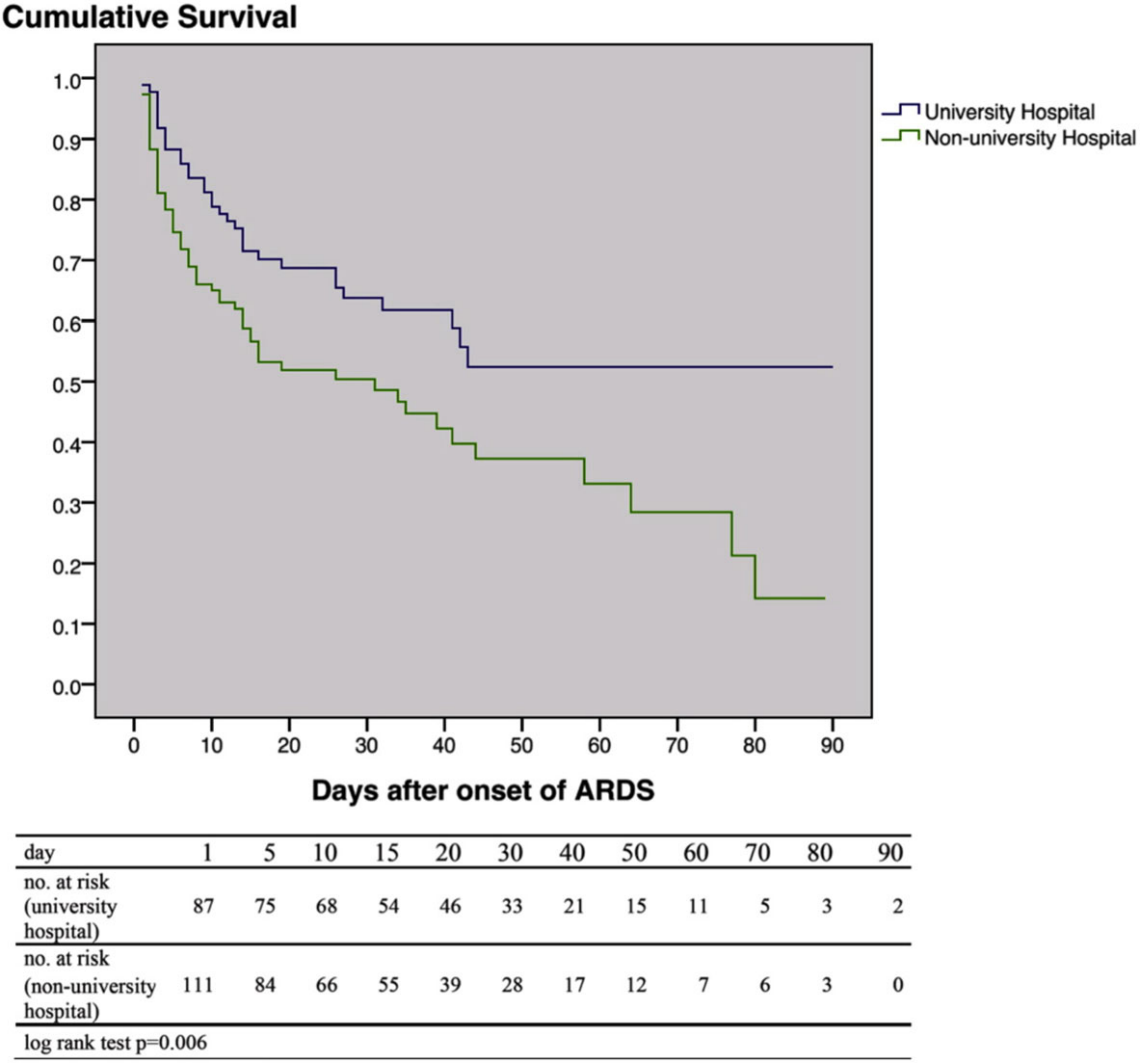
Probability of survival of mechanically ventilated patients after the development of ARDS, in university and non-university hospitals (after Kaplan–Meier). *ARDS* acute respiratory distress syndrome, *no.* number

Of all ventilated patients, 19.3% (2.6% of all ICU admissions) developed ARDS (*n* = 198). Patients with ARDS developed more complications and their hospital mortality (49.5%) was double that of patients without ARDS (Fig. 1, Additional file 1: Tables S1 and S2). For ARDS patients, hospital mortality was 18.2% higher in non-university hospitals than in university hospitals; moreover, even ventilated patients without ARDS had higher mortality (i.e. 9.6%) in non-university hospitals (Figs. 1 and 2).

Demographic characteristics, illness severity, and severity of lung injury in ARDS patients were similar in university and non-university hospitals, whereas late ARDS and complications until onset of ARDS occurred more frequently in the university hospitals (Table 1). Independent from the baseline characteristics and from complications occurring later during ventilation, treatment in a non-university hospital was an important independent risk factor for increased hospital mortality in patients with ARDS (Table 2), but not in patients without ARDS (Additional file 1: Table S3). Although each hospital was characterized in detail by the setting of care variables, these factors did not contribute to the increased mortality risk. This increased risk in non-university hospitals was confirmed by an additional independent analysis, in which patient groups were compared between hospitals, and potential confounding factors at onset of ARDS were selected clinically and statistically controlled for. This one-step multivariable analysis showed similar and significant results, even when one or various combinations of factors controlled for were excluded in the sensitivity analyses (Table 3).

**Table 2:** Univariable and multivariable analysis of factors associated with hospital mortality in ventilated patients with ARDS

Variable	Univariable analysis^a^		Multivariable analysis^b^	
	Odds ratio (95% CI)	*p* value	Odds ratio (95% CI)	*p* value
Factors related to patients’ baseline factors				
Sex		0.068		
Male	1.82 (0.96–3.46)			
Female	1			
SAPS II at ICU admission		0.005		
≤40	1			
40–59	2.09 (1.03–4.22)			
≥60	3.69 (1.64–8.29)			
Factors related to individual patient management				
FiO_2_ (0.1 increments)	1.79 (1.35–2.38)	<0.001	1.62 (1.17–2.24)	0.004
Driving pressure^c^ (1 cmH_2_O increments)	1.17 (1.08–1.26)	<0.001	1.10 (1.02–1.20)	0.016
Use of vasoactive drugs (2 consecutive days)	2.01 (1.00–4.02)	0.049		
Factors developing during mechanical ventilation				
Liver failure	7.85 (2.24–27.55)	0.001	6.93 (1.68–28.59)	0.007
Renal failure	4.15 (2.25–7.64)	<0.001	3.46 (1.59–7.55)	0.002
Metabolic acidosis	3.29 (1.48–7.29)	0.003	2.94 (1.03–8.27)	0.043
pHa		0.001		
Acidosis (<7.35)	4.09 (1.90–8.77)			
7.35–7.45	1			
Alkalosis (>7.45)	1.17 (0.55–2.47)			
Lowest PaO_2_/FiO_2_ (mmHg)		0.009		
≤100	3.08 (1.38–6.85)			
100–150	2.53 (1.26–5.10)			
>150	1			
Factors related to the setting of care				
Non-university hospital	2.10 (1.17–3.75)	0.013	2.89 (1.31–6.38)	0.008
University hospital	1		1	

*ARDS* acute respiratory distress syndrome, *CI* confidence interval, *SAPS II* Severe Acute Physiology Score II, *ICU* intensive care unit, *FiO*_*2*_ fraction of inspired oxygen, *PaO*_*2*_ partial pressure of arterial oxygen tension, *PEEP* positive end-expiratory pressure

^a^Only those variables are shown that qualified with *p* < 0.1 in the four prior multivariate analyses within the four variable categories for the overall final multivariable model (as described in Methods and in Additional file 1, the other variables in each category including tidal volume, PEEP, hospital size, ICU size and case volume of ventilated patients per ICU were eliminated in the multivariate analysis within each category by the presented parameters and did not qualify for the final model)

^b^Only those variables are shown that remained significant in the final multivariable model for *n* = 169 with stepwise backward elimination using a threshold of *p* = 0.05 according to Wald statistics. Goodness of fit: Hosmer–Lemeshow test, *p* = 0.398; area under the receiver-operator curve, 0.84 (95% CI 0.78–0.90), *p* < 0.001

^c^Driving pressure = plateau pressure – PEEP

**Table 3:** Control for confounding and sensitivity analyses with a further multivariable model including only potential confounders at ARDS onset

	Odds ratio for hospital mortality of ARDS patients in non-university hospitals vs university hospitals		
	*n* ^a^	Odds ratio multivariable	*p* value
Multivariable analysis with all potential confounders	128	2.8 (1.1–7.1)	0.035
Excluding SAPS II	137	2.7 (1.1–6.6)	0.032
Excluding BMI	142	3.5 (1.5–8.4)	0.004
Excluding compliance	151	2.4 (1.1–5.5)	0.037
Excluding SAPS II, BMI	151	3.4 (1.5–7.7)	0.005
Excluding compliance, SAPS II	164	2.4 (1.1–5.4)	0.028
Excluding compliance, BMI	174	3.5 (1.7–7.6)	0.001
Excluding compliance, SAPS II, BMI	189	3.3 (1.6–6.7)	0.001

The consistency of results of these sensitivity analyses indicates that this multivariable model including only potential confounders was robust with respect to alterations of included variables or to the corresponding changes of the study group

To control for confounding only potential confounders were considered in this further multivariate model, including factors that differ between ARDS patients in university and non-university hospitals at onset of ARDS and also including those factors that did not differ significantly but only numerically between groups (Table 1): age, BMI, SAPS II, main problem (medical/surgical), ARDS origin (pulmonary/extrapulmonary), day of ARDS onset, dynamic compliance at ARDS onset, reason for initiation of mechanical ventilation, (postoperative acute respiratory failure (*ARF*), ARF after aspiration, ARF after multiple trauma), and comorbidities or complications present until onset of ARDS (sepsis, pneumonia, cardiovascular failure, coagulation failure, liver failure, metabolic acidosis)

*ARDS* acute respiratory distress syndrome, *SAPS II* Severe Acute Physiology Score II, *BMI* body mass index

^a^Number of ARDS patients included in the respective multivariable analysis after excluding factors or various combinations of factors with missing values (SAPS II 5% missing values, BMI 7%, compliance 12%) (all ARDS patients *n* = 198, eight patients without outcome variable, one patient without age)

**Fig. 3:**
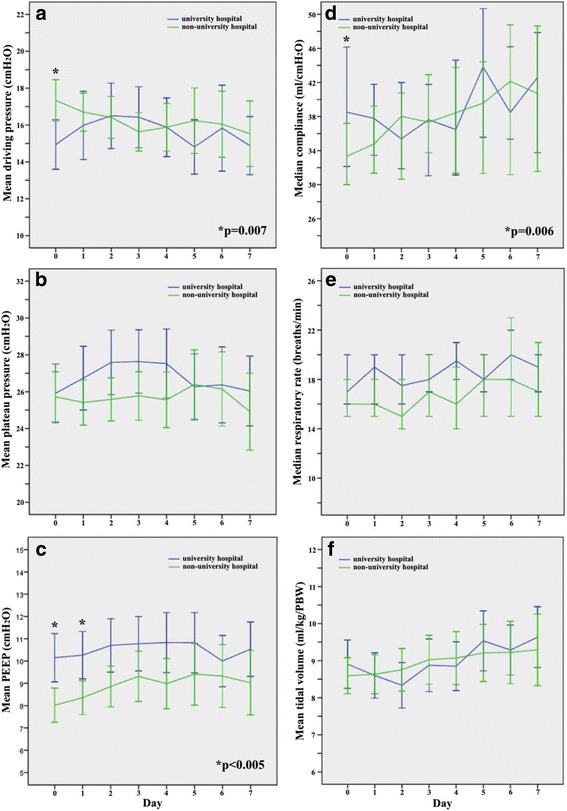
Change over time in ventilatory parameters during the first week after onset of ARDS. **a** Driving pressure (= plateau pressure – PEEP), **b** plateau pressure, **c** PEEP, **d** compliance (= tidal volume / (plateau pressure – PEEP)), **e** respiratory rate, **f** tidal volume/kg predicted body weight. *Error bars* show 95% confidence intervals. *Differences between university and non-university hospitals during the day after onset of ARDS (mean ± SD): driving pressure, 14.9 ± 5.6 vs 17.3 ± 5.5 cmH_2_O, *p* = 0.007; PEEP, 10.2 ± 5.1 vs 8.0 ± 4.1 cmH_2_O, *p* = 0.002; compliance, 47.9 ± 28.0 vs 36.8 ± 19.9 ml/cmH_2_O, *p* = 0.006. PEEP also differed between university and non-university hospitals during the second day after the onset of ARDS: 10.3 ± 4.9 vs 8.4 ± 4.0 cmH_2_O, *p* = 0.004. *PEEP* positive end-expiratory pressure

**Fig. 4:**
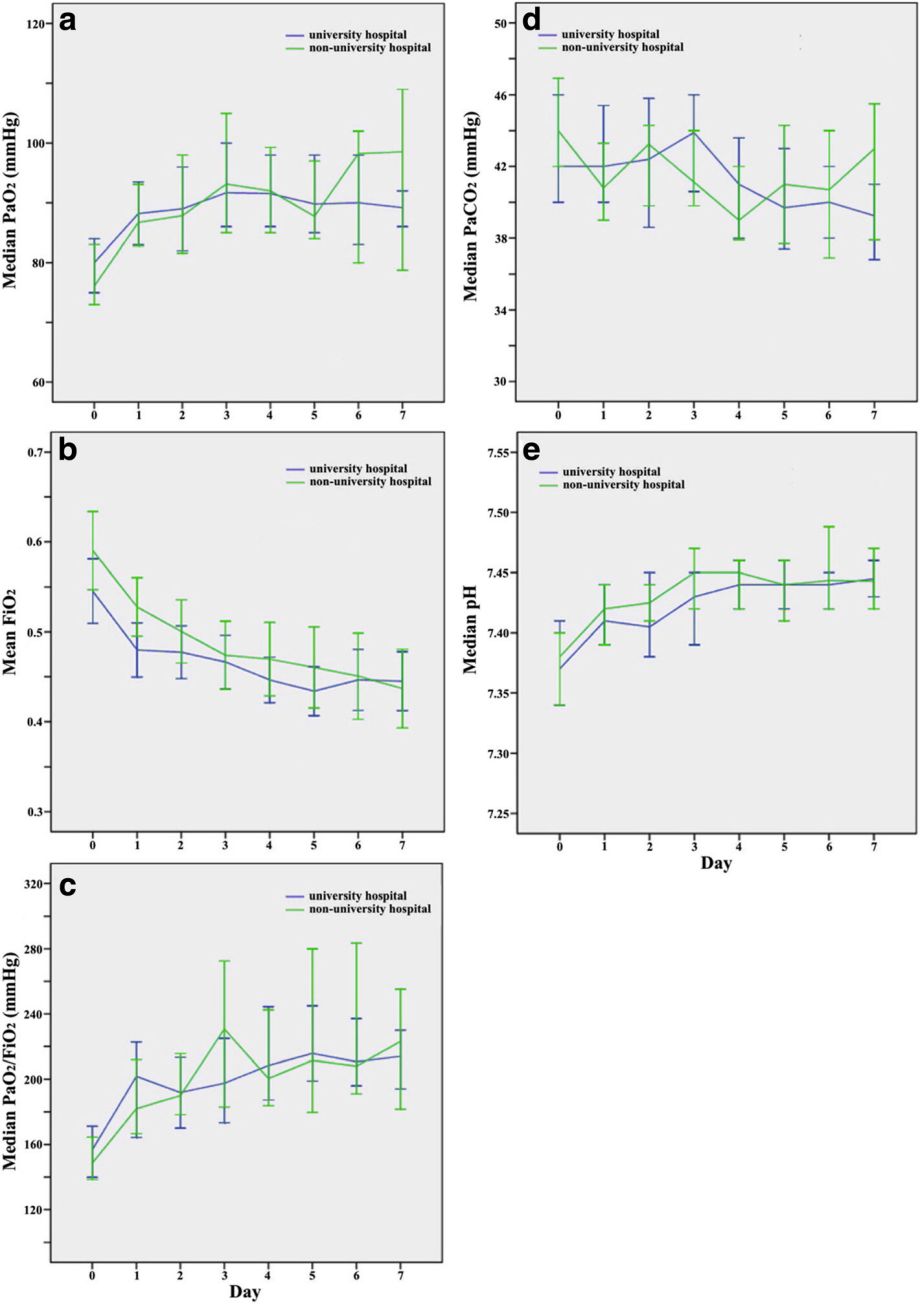
Change over time in gas exchange parameters and pH after onset of ARDS. **a** Partial pressure of arterial oxygen tension (*PaO*_*2*_), **b** fraction of inspired oxygen (*FiO*_*2*_), **c** arterial-to-inspired-oxygen (*PaO*_*2*_ */FiO*_*2*_), **d** partial pressure of arterial carbon dioxide tension (*PaCO*_*2*_), **e** pH. *Error bars* show 95% confidence intervals

**Fig. 5:**
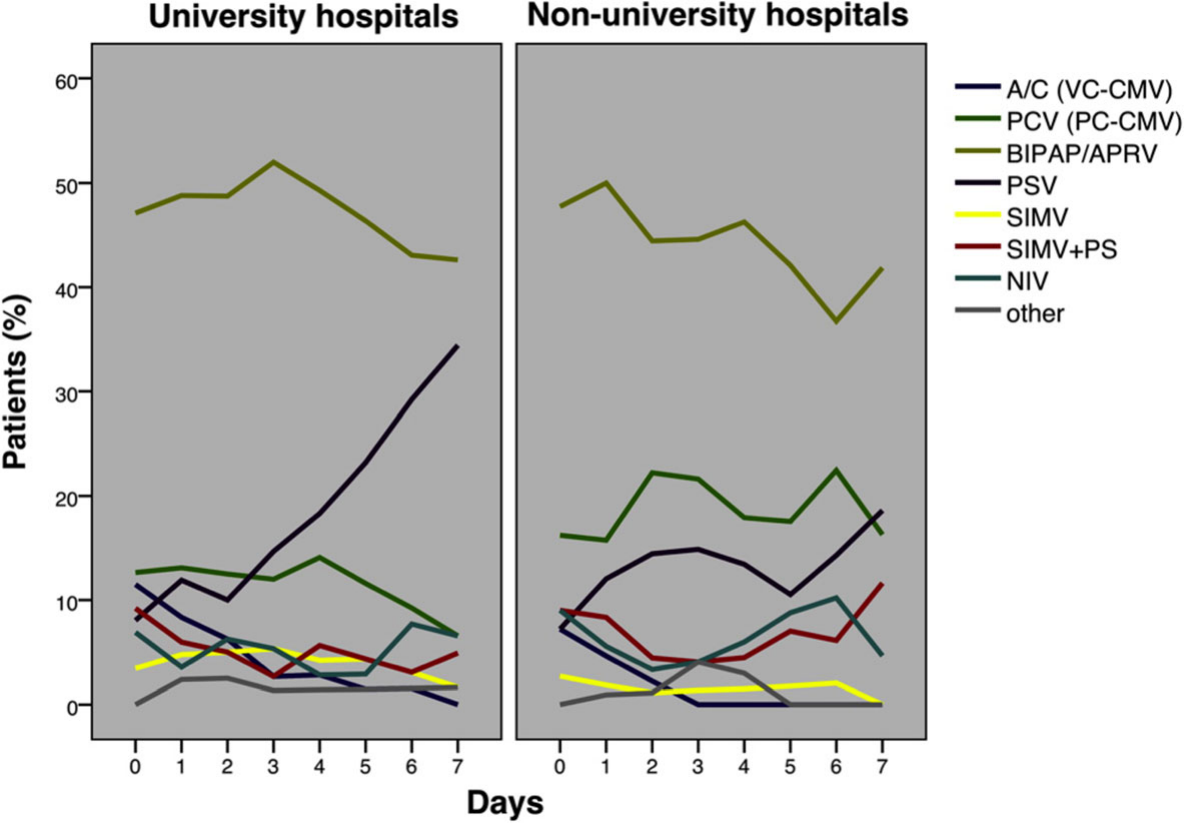
Ventilatory modes used during the first week after the onset of ARDS in patients in university and non-university hospitals. Pressure support ventilation was used both more often and earlier in university hospitals than in non-university hospitals (see Table 6 for *p* values). *A/C (VC-CMV)* denotes assist/control (volume-controlled continuous mechanical ventilation), *PCV (PC-CMV)* pressure-controlled ventilation (pressure-controlled continuous mechanical ventilation), *BIPAP/APRV* biphasic positive airway pressure/airway pressure release ventilation, *PSV* pressure-support ventilation, *SIMV* synchronized intermittent mandatory ventilation, *NIV* non-invasive ventilation

Of all factors related to individual patient management, only the driving pressure and FiO_2_ were identified as independent risk factors in ARDS patients, with a significantly increasing mortality risk of 10% for every 1 cmH_2_O increment of driving pressure and 62% for every 0.1 increment of FiO_2_ (Table 2). Most of the ventilatory and gas exchange parameters were similar between the hospitals. However, during the first day after ARDS onset, and also during therapeutic ventilation in university hospitals, ARDS patients were ventilated with higher PEEP and lower driving pressure whereas there was only a trend for a lower FiO_2_ (Figs. 3 and 4, Table 4). Organ failure was also independently associated with mortality (Table 2); however, the incidence of organ failure and complications after onset of ARDS was considerably higher in the university hospitals (Table 5). Prone positioning, neuromuscular blocking, and volume-controlled ventilation were rarely used, whereas pressure-controlled ventilatory modes (mainly biphasic positive airway pressure (BIPAP)) and pressure support ventilation were applied for ≥75% of the ventilation days (Fig. 5, Table 4). ARDS patients underwent tracheostomy twice as frequently and more often percutaneously in university hospitals, whereas there were no differences in other weaning characteristics (Table 6). In university hospitals, ventilation and length of stay in the ICU of ARDS patients lasted about twice as long (Table 6), and fewer patients per ICU bed but more than twice as many ventilated patients per ICU were treated (Table 7).

**Table 4:** Ventilatory characteristics and adjunctive therapies during the first week after ARDS onset in university and non-university hospitals

	University hospital (*n* = 87; 592 days of mechanical ventilation)	Non-university hospital (*n* = 111; 599 days of mechanical ventilation)	*p* value
Ventilatory parameters averaged over the first week after ARDS onset, mean (SD); median (IQR)			
Driving pressure^a^ (cmH_2_O)	15.7 (5.1); 15 (13–19)	16.3 (4.7); 16 (13–19)	0.40
Driving pressure when FiO_2_ > 0.4 and PEEP ≥ 5 (cmH_2_O)^b^	15.1 (4.4); 16 (12–19)	17.0 (5.0); 18 (14–20)	0.02
PEEP (cmH_2_O)	10.1 (4.2); 10 (7–13)	8.4 (3.9); 8 (6–11)	0.005
PEEP when FiO_2_ > 0.4 and PEEP ≥ 5 (cmH_2_O)^b^	11.7 (4.7); 10 (8–15)	9.7 (3.7); 9 (7–12)	0.005
PEEP categories, *n* (%)			
≤5 cmH_2_O	11 (12.6)	24 (21.8)	0.05
≤10 cmH_2_O	41 (47.1)	58 (52.7)	
≥11 cmH_2_O	35 (40.2)	28 (25.5)	
Tidal volume (ml/kg PBW)	8.9 (2.0); 8.6 (7.6–10.0)	8.9 (2.4); 8.7 (7.6–10.0)	0.99
Plateau pressure (cmH_2_O)	26.4 (5.8); 27 (23–31)	25.3 (5.4); 25 (22–28)	0.18
Plateau pressure categories, *n* (%)			
≤30 cmH_2_O	54 (70.1)	89 (86.4)	0.007
>30 cmH_2_O	23 (29.9)	14 (13.6)	
Compliance^c^ (ml/mbar; ml/mbar/kg PBW)	42 (32–54); 0.62 (0.49–0.80)	38 (29–48); 0.59 (0.45–0.73)	0.06; 0.23
Respiratory frequency (breaths/min)	20 (16–22)	18 (13–22)	0.17
FiO_2_	0.44 (0.40–0.54)	0.48 (0.41–0.57)	0.07
PaO_2_ (mmHg)	91 (84–105)	92 (78–107)	0.64
PaO_2_/FiO_2_ (mmHg)	208 (169–250)	194 (155–248)	0.19
PaCO_2_ (mmHg)	42 (38–47)	42 (37–48)	0.91
pHa	7.41 (7.36–7.45)	7.42 (7.36–7.46)	0.59
Days with ventilatory mode during the first week after ARDS onset, *n* (% of all days of mechanical ventilation)			<0.001
Biphasic intermittent positive airway pressure	281 (47.5)	271 (45.2)	0.44
Pressure controlled ventilation	69 (11.7)	111 (18.5)	0.001
Pressure support ventilation	105 (17.7)	75 (12.5)	0.01
SIMV + pressure support ventilation	31 (5.2)	41 (6.8)	0.24
SIMV	24 (4.1)	10 (1.7)	0.01
Volume-controlled ventilation (assist/control)	28 (4.7)	15 (2.5)	0.04
Non-invasive ventilation	31 (5.2)	38 (6.3)	0.41
Other	9 (1.5)	7 (1.2)	0.60
Not specified	14 (2.4)	31 (5.2)	0.01
Days applying adjunctive therapies, *n* (% of all days of mechanical ventilation); median (IQR)			
Prone positioning or rotation bed, all days	44 (7.4)	22 (3.7)	0.005
Sedatives all days, duration/patient (days)	439 (74.2); 12 (7–21)	411 (56.8); 5 (2–10)	0.03; <0.001
Neuromuscular blocking agents, all days	11 (1.9)	7 (1.2)	0.33

*ARDS* acute respiratory distress syndrome, *FiO*_*2*_ fraction of inspired oxygen, *PaO*_*2*_ partial pressure of arterial oxygen tension, *PaO*_*2*_ */FiO*_*2*_ arterial-to-inspired oxygen ratio, *PEEP* positive end-expiratory pressure, *PBW* predicted body weight, *SIMV* Synchronized intermittent mandatory ventilation, *SD* standard deviation, *IQR* interquartile range

^a^Driving pressure = plateau pressure – PEEP

^b^All other ventilatory parameters during therapeutic ventilation did not differ between groups in this sensitivity analysis, including only those ventilation days – during the first week after ARDS onset with FiO_2_ > 0.4 and PEEP ≥ 5 cmH_2_O – that represented minimum values as criteria for the start of weaning in the low tidal volume trial of the ARDS Network [12] (261 of 592 days (44.1%) in university hospitals and 299 of 599 days (49.9%) in non-university hospitals)

^c^Compliance = tidal volume / (plateau pressure – PEEP)

**Table 5:** Incidence of complications developing after ARDS onset in university and non-university hospitals

	Incidence			Incidence rate	
	University hospital (*n* = 87)	Non-university hospital (*n* = 111)		University hospital (*n* = 87)	Non-university hospital (*n* = 111)
	*n* ^a^ (%)	*n* ^a^ (%)	*p* value	Rate/1000 days^b^ (analyzed days^c^)	Rate/1000 days^b^ (analyzed days^c^)
Sepsis	8/46 (17.4)	3/73 (4.1)	0.02	12 (671)	5 (653)
Pneumonia	17/44 (38.6)	13/67 (19.4)	0.03	30 (570)	21 (609)
Cardiovascular failure	11/24 (45.8)	12/40 (30)	0.20	38 (289)	28 (423)
Liver failure	7/76 (9.2)	3/109 (2.8)	0.09	6 (1139)	3 (972)
Renal failure	15/61 (24.6)	11/79 (13.9)	0.11	16 (942)	15 (755)
Coagulopathy	13/64 (20.3)	8/103 (7.8)	0.02	13 (966)	9 (916)
Metabolic acidosis	7/79 (8.9)	8/98 (8.2)	0.87	6 (1159)	9 (911)
Respiratory acidosis	16/69 (23.2)	9/88 (10.2)	0.03	17 (995)	11 (795)
Barotrauma	2/23 (2.7)	0/98 (0)	0.18	2 (1099)	0 (849)

*ARDS* acute respiratory distress syndrome

^a^Number of patients who developed this complication after the first day of ARDS onset as a proportion of those patients without this complication until ARDS onset

^b^Incidence rate of the complication after the first day of ARDS onset per 1000 days of mechanical ventilation in those patients without this complication until ARDS onset

^c^Remaining days for analysis of a total of 1249 days of mechanical ventilation after ARDS onset in university hospitals and 988 days in non-university hospitals (observation only during mechanical ventilation and up to 28 days after initiation of mechanical ventilation)

**Table 6:** Characteristics of weaning and duration of mechanical ventilation, ICU and hospital stay in patients with ARDS

	University hospital (*n* = 87)	Non-university hospital (*n* = 111)	*p* value
Tracheostomy			0.16
Tracheostomy after ARDS onset/nontracheostomized patients until ARDS onset	33/76 (43.4)	20/102 (19.6)	0.001
Tracheostomy, percutaneous /surgical/not specified	29 (87.9)/3 (9.1)/1 (3.0)	11 (55.0)/8 (40.0)/1 (5.0)	0.01
Days to tracheostomy after ARDS onset	10 (4–13)	4 (2–11)	0.10
Main weaning method			0.16
Daily spontaneous weaning trial	12 (19.7)	25 (34.7)	
Gradual reduction of ventilatory support	45 (73.8)	43 (59.7)	
Not specified	4 (6.6)	4 (5.6)	
Extubation			
Extubation	42/86 (48.8)	63/107 (58.9)	0.16
Reintubation within 48 h	17/42 (40.5)	16/53 (25.4)	0.10
Non-invasive ventilation after extubation; reintubation thereafter	23/42 (54.8); 10 (43.5)	27/63 (42.9); 12 (44.4)	0.23; 0.95
Duration of mechanical ventilation (days)	16 (9–29)	8 (3–16)	<0.001
After ARDS onset	13 (6–23)	6 (3–14)	<0.001
Survivors	14 (9–25)	7 (3–14)	<0.001
Length of ICU stay (days)	19 (12–34)	10 (5–23)	<0.001
After initiation of mechanical ventilation	19 (11–34)	10 (5–24)	<0.001
After ARDS onset	15 (9–31)	8 (4–18)	<0.001
Survivors	19 (13–35)	13 (5–24)	0.003
Length of hospital stay (days)	31 (17–46)	21 (9–41)	0.007
After initiation of mechanical ventilation	26 (14–42)	15 (6–34)	0.002
After ARDS onset	23 (11–37)	14 (4–28)	0.005
Survivors	32 (19–53)	24 (14–41)	0.077

Data presented as *n* (%); median (interquartile range)

*ARDS* acute respiratory distress syndrome, *ICU* intensive care unit

**Table 7:** Setting of care in university and non-university hospitals

	University hospital	Non-university hospital	*p* value
Number of hospitals	18 (22.5)	62 (77.5)	
Number of participating ICUs	33 (34.7)	62 (65.3)	
Beds per hospital	1275 (1189–1444)	367 (280–526)	<0.001
Beds per ICU	13 (10–16)	9 (7–12)	0.001
ICU size category			
< 5/<8/ <14/ ≥14 beds per ICU	4 (12.1)/3 (9.1)/9 (27.3)/17 (51.5)	18 (29)/21 (33.9)/18 (29)/5 (8.1)	<0.001
Total number of all ICU beds	465 (43.2)	612 (56.8)	
ICU specialization			<0.001
Surgical/medical	6 (18.2)	41 (66.1)	
Medical	8 (24.2)	6 (9.7)	
Surgical	16 (48.5)	15 (24.2)	
Neurological	3 (9.1)	0	
Number of all ICU patients	2994 (39.7)	4546 (60.3)	
Patients per ICU	74 (51–106)	74 (49–98)	0.47
Patients per ICU bed	5.8 (4.4–8.0)	7.7 (5.2–9.6)	0.04
Number of ventilated patients	558 (54.3)	470 (45.7)	
Ventilated patients per ICU	14 (8–23)	6 (4–10)	<0.001
Ventilated patients per ICU bed	1.0 (0.7–1.5)	0.7 (0.5–1.0)	0.002
% ventilated patients in all ICUs	19 (12–27)	10 (6–15)	<0.001

Data presented as *n* (%) or median (interquartile range)

*ICU* intensive care unit

## Discussion

Hospital mortality of ARDS patients in non-university hospitals was considerably higher than in university hospitals; moreover, treatment in non-university hospitals was an important risk factor in ARDS patients, increasing the mortality risk to an extent similar to that of renal failure, independent of illness severity, the occurrence of organ failure, or other variables. Although the study design was observational and, therefore, the two groups were not compiled by randomization, the remarkable difference in mortality was not due to differences between the groups; that is, in both groups patients were very similar with respect to important demographic and clinical criteria (e.g., age, SAPS II, underlying disease, and PaO_2_/FiO_2_ at onset of ARDS). Even though the groups could not be equal based on the study design, when considering the predefined potential confounders at the onset of ARDS, the difference in mortality risk between university and non-university hospitals was still significant and substantial. Our study seems to be the first to provide statistically robust evidence that ARDS treatment in non-university hospitals is associated with higher mortality compared with university hospitals.

Decades ago, Lachmann [26, 27] suggested using ventilation modes that allow the lowest possible driving pressure in an open ARDS lung in order to prevent lung damage due to high shear forces between open and closed lung units. Driving pressure and FiO_2_, but neither PEEP nor other ventilatory variables, were independently associated with increased mortality in ARDS patients. Increasing driving pressure and FiO_2_ reflect more severe stages of ARDS but also further aggravate lung injury [26]. Although increasing PEEP also reflects more severe stages of ARDS, low values increase mortality (as opposed to driving pressure and FiO_2_) by further aggravating lung injury [15, 23, 26, 28]. This nonlinear relationship between mortality and PEEP in ARDS may explain that neither increasing nor decreasing PEEP was associated with increasing mortality in our multivariable analysis. However, adequate PEEP prevents atelectasis, alveolar flooding, and collapse and is therefore a precondition for both lower driving pressures and FiO_2_ by maintaining lung volume and area for gas exchange in ARDS [5, 23]. Then again, low PEEP results in higher driving pressures (due to less aerated lung volume), but also in higher FiO_2_ to maintain oxygenation with a smaller area for gas exchange [15–22, 24, 26]. Accordingly, as in the present analysis, in the multivariable analysis performed by Ferguson et al. [23] of ARDS patients in the First VENTILA study, increases in FiO_2_ (and not decreases in PaO_2_/FiO_2_), with very similar ORs, were independently associated with mortality.

Driving pressure was not included in this analysis by Ferguson et al. [23], or in that of the KCLIP study [22] or in other multivariable analyses performed in ARDS patients [9, 12, 14], with the exception of the recent analysis by Amato et al. [24]. These latter authors analyzed nine randomized ARDS trials (including 17-year-old data) and, as in the present analysis, also found that both driving pressure and FiO_2_ were significantly associated with survival [24]. Our analysis is the first to confirm their findings and, surprisingly, our results are very similar despite the use of different methods (e.g., Amato et al. used the mean driving pressure of the first 24 h after randomization and we used the mean value of the first week after onset of ARDS in an observational study). In the present study, the OR that indicates increased mortality risk with higher driving pressure for ARDS patients in university hospitals corresponds to a very similar relative risk when compared with Amato et al. [24] (Additional file 1: Figure S1).

Although any comparisons between studies across national borders must be interpreted with considerable caution, the increased mortality found in German non-university hospitals was not excessively high but was very similar to other observational studies (despite higher age and SAPS II) [9, 10]; in contrast, despite SAPS II being 13 points higher, mortality of ARDS in non-university hospitals was 5.7% lower compared with the other 22 countries of the Second VENTILA study [7]. Therefore, in the present study, the considerable difference in mortality between the university and non-university hospitals cannot be attributed to exceedingly high mortality in the non-university hospitals but rather to the relatively low mortality rate of 39.3% in the university hospitals (being 8.5% lower than that in the ALIEN study [11]).

In ventilated patients, organ failure is associated with increased mortality [6, 12, 14] and this was also the case in the present analysis. Surprisingly, the outcome of ARDS patients in university hospitals was better even though organ failure and complications occurred considerably more often, even when taking into account different observation periods by comparing incidence rates rather than proportions. Finally, because the difference in mortality was not related to organ failure, complications, illness severity, or patient characteristics, the unequal outcome of ARDS is most likely related to differences in treatment practices. Ventilatory modes and adjunctive therapies did not differ sufficiently to really contribute to the unequal mortality, and there was only a trend for use of a higher FiO_2_ in non-university hospitals. Tidal volumes and plateau pressures also did not differ between the groups, but PEEP was higher and driving pressure was lower in university hospitals during therapeutic ventilation. In the German university hospitals, driving pressures of about 15 cmH_2_O were similar to those in the ALIEN study [11] and in the meta-analysis performed by Briel et al. [28], despite tidal volumes being higher to the extent of 2 and 2.6 ml/kg predicted body weight, respectively. However, in contrast to the present analysis, higher PEEP did not lead to lower driving pressures in the meta-analysis by Briel et al., and the authors found a difference in mortality rate of only 5% [28]. In our study, because higher driving pressures and FiO_2_ were independently associated with increased mortality, and because higher PEEP is the precondition to reduce both variables, the differences in ventilatory therapy between university and non-university hospitals may partially explain the unequal outcome of ARDS patients.

In the university hospitals, the duration of ventilation and ICU stay was similar to that in the ALIEN study [11] and twice as high as that in non-university hospitals. The higher rate of complications in university hospitals may be a result of, but also a contributory cause of, longer ventilation. Non-invasive ventilation after extubation, reintubation rates, timing of tracheostomy, and other weaning characteristics did not differ between groups. However, in the university hospitals, tracheostomy was performed twice as frequently and more often percutaneously, and this is reported to be associated with a lower risk of wound infection than a surgical tracheostomy [29]. The difference in ICU mortality between university and non-university hospitals was not significant, and only half that of the difference in overall hospital mortality. Although the reason for this considerably increased difference in mortality after ICU discharge remains unknown, a possible explanation could be that in non-university hospitals the ventilatory therapy was too short and discharge from the ICU too early for some of the patients. This may lead to reconsideration about the sufficient duration of ventilation and ICU therapy when patients develop ARDS, rather than focusing on surrogate outcomes such as ventilator-free days [17, 18].

In the ICUs of the university hospitals more than twice as many ventilated patients per ICU were treated compared with non-university hospitals; however, the percentage of ventilated patients was still considerably less compared with other countries [6, 7]. Kahn et al. [30] demonstrated that the high case volume of ventilated patients is independently associated with better survival in nonsurgical patients. This annualized hospital volume cannot be directly compared with our case volume of ventilated nonsurgical and surgical patients per ICU, because most of our hospitals had various ICUs that did not participate in the present study; therefore, our ICU volume does not fully represent hospital volume. In patients without ARDS, this ICU volume was an independent risk factor with a significantly decreasing mortality risk of 18% for every increment of 10 ventilated patients per month. In patients with ARDS, the ICU volume was not independently associated with mortality, which does not prove a missing association between case volume and mortality. On the other hand, some ICUs with a very low case volume of ventilated patients were included and this is likely to be an important factor.

Apart from mechanical ventilation, many other aspects of treatment are important for the outcome of these severely ill patients, and these items may also differ between hospitals. This includes, in particular, the effective and perhaps more time-consuming multidisciplinary treatment of both underlying diseases and complications, the presence of trained physicians and intensivists, an adequate nurse-to-patient ratio and staff workload, the implementation of protocols, as well as other factors that were not assessed in the Second VENTILA study [13, 31–33].

### Limitations

The idea for this present analysis came rather late and took a considerable amount of time, and the data are now more than 10 years old. However, due to an increasing lack of physicians, many non-university hospitals included in the present analysis did not participate in the more recent VENTILA studies [8]; therefore, these data from 2004 are still the best data available. Moreover, this is the last study undertaken before extracorporeal lung assist devices were widely introduced in Germany, with a possible impact on outcome [34]. However, it is unknown whether our findings can be extrapolated to other countries and to what extent they represent the current state of therapy in Germany. In addition, the enormous difference in the duration of ventilation which goes in the opposite direction to mortality, and the extremely high number of reintubations in both university and non-university hospitals, may also indicate that considerable caution is required when transferring our data to other settings. Tidal volumes were high and did not differ between the groups but non-university hospitals used less lung protective ventilation; this might suggest slower implementation of lung protective strategies at that time compared with university hospitals, whereas this might not be true today. Nevertheless, the Large Observational Study to Understand the Global Impact of Severe Acute Respiratory Failure (LUNG SAFE) showed that, in 2014, there were still major problems with the diagnosis of ARDS and the implementation of lung protective ventilation [35]. Potential bias due to selection is a further limitation of our analysis as we included only those hospitals that volunteered to participate. Nevertheless, the 80 participating hospitals were located throughout Germany and non-university hospitals were not underrepresented as compared with other observational studies [9–13] but, conversely, constituted ≥75% of the participating hospitals thereby including ≥50% of the ARDS patients.

## Conclusions

The care setting had an important impact on ARDS outcome, as the mortality risk in non-university hospitals was sometimes even tripled compared with that in university hospitals. Differences in ventilatory care included: a 2 cmH_2_O higher PEEP combined with a 2 cmH_2_O lower driving pressure; ventilation that lasted twice as long in university hospitals; and the case volume of ventilated patients per ICU was more than doubled in university hospitals compared with that in non-university hospitals. The combination of lower driving pressure with higher PEEP, and other approaches to reduce both driving pressure and FiO_2_, represent prognostically relevant treatment goals in ARDS that are exportable to non-university hospitals. In addition, our results may also indicate the need to further examine those practices common in university hospitals that favor better outcome in ARDS, as they may be transferable to non-university hospitals.

## Appendix

Participating investigators (in alphabetic order of cities: city, name of the hospital and investigators): **Aachen**, Katholische Stiftung Marienhospital Aachen, T. Möllhoff, K. Tsompanidis; **Aachen**, Universitätsklinikum Aachen, D. Henzler, R. Kuhlen; **Andernach**, St. Nikolaus-Stiftshospital GmbH, W. Baier; **Bad Salzungen**, Klinikum Bad Salzungen gGmbH, A. Lunkeit, J. Schlechtweg; **Bad Urach**, Ermstalklinik Bad Urach, V. Tumbass, S. Hahn; **Baden-Baden**, Stadtklinik Baden-Baden, V. Schöffel, K. van Deyk, S. Seyboth; **Berlin**, Charité Universitätsklinikum, Campus Mitte und Campus Virchow, S. Weber-Carstens, K. Haid, C. Melzer-Gartzke, C. von Heymann, B. Temmesfeld, M. Oppert, S. Rosseau; **Berlin**, Vivantes Krankenhaus am Urban, Kreuzberg, H.J. Hartung; **Berlin**, Vivantes-Klinikum Neukölln, H. Gerlach; **Berlin**, Parkklinik Weißensee Berlin, Goldstein; **Berlin**, Lungenklinik Heckeshorn, M. Reffenberg; **Bielefeld**, Städtische Kliniken Bielefeld gGmbH, J. Kleideiter, P. Palomino; **Bonn**, Universitätsklinikum Bonn, H. Wrigge, C. Putensen, F.L. Dumoulin; **Bonn**, Evangelisches Waldkrankenhaus Bad Godesberg gGmbH, M. Födisch, J. Busch; **Bovenden-Lenglern**, Evangelisches Krankenhaus Göttingen-Weende e.V., K. Schild, C.-P. Criée; **Chemnitz**, Zeisigwaldkliniken Bethanien Chemnitz, B. Albrecht; **Cloppenburg**, St. Josefs-Stift Cloppenburg, C. Weilbach, M. Raab; **Cottbus**, Carl-Thiem-Klinikum Cottbus gGmbH, R. Wittich; **Darmstadt**, Evangelisches Krankenhaus Elisabethenstift gGmbH, J. Buettner; **Dinslaken**, Evangelisches und Johanniter Klinikum, H. Militzer; **Dormagen**, Kreiskrankenhaus Dormagen, E. Schröder, F.L. Deres; **Dresden**, Krankenhaus Dresden-Friedrichstadt, P. Kern, A. Nowak, T. Pahlitzsch, K.F. Rothe; **Dresden**, Universitätsklinikum Carl Gustav Carus Dresden, M. Ragaller, T. Koch; **Ebersbach**, Kreiskrankenhaus Löbau, G. Sterzel; **Eberswalde**, Klinikum Barnim GmbH, Werner Forßmann Krankenhaus, C. Werel, A. Kopietz; **Essen**, Universitätklinikum Essen, M. Beiderlinden; **Essen**, Ruhrlandklinik, H. Steiniger, V. Weißkopf; **Freiburg im Breisgau**, Evangelisches Diakoniekrankenhaus, H. Kerger, J. Ernst; **Gauting**, Asklepios Fachkliniken München-Gauting, O. Karg; **Göppingen**, Klinik am Eichert Göppingen, H. Mende, M. Fischer, J. Martin, A. Aßmann; **Goslar**, Dr. Herbert-Nieper-Krankenhaus-Goslar, J. Heine, M. Borth, U. von Leitner, M. Hoffmann; **Göttingen**, Universitätsklinikum der Georg-August-Universität Göttingen, M. Quintel; **Halle/Saale**, Universitätsklinikum der Martin-Luther-Universität Halle-Wittenberg, H. Bromber, J. Soukup, G. Leonhardt; **Halle/Saale**, Krankenhaus St. Elisabeth und St. Barbara Halle (Saale), C. Wuttke; **Halle/Saale**, Städtisches Krankenhaus Martha-Maria Halle-Dölau gGmbH, M. Holler; **Hamburg**, Allgemeines Krankenhaus Harburg, G. Savinski, Thomas Klöss; **Hameln**, Kreiskrankenhaus Hameln, D. Korth, W. Seitz; **Hannover**, Klinikum Hannover Nordstadt, S. Krueper; **Hannover**, Medizinische Hochschule Hannover, J. Ahrens, U. Molitoris, K. Johanning, K. Raymondos; **Heidelberg**, Universitätsklinikum der Ruprecht-Karls-Universität Heidelberg, J.F. Meyer; **Heidelberg**, Thoraxklinik Heidelberg gGmbH, M. Layer; **Kamen**, Städt. Hellmig-Krankenhaus, Eller; **Köln**, Kliniken der Stadt Köln Krankenhaus Holweide, F. Ragaymutu; Leipzig, St. Elisabeth gGmbH Leipzig, K. Rudolph, J. Raumanns; **Leipzig**, Bundeswehrkrankenhaus Leipzig, U. Grüneisen, F. Stupacher; **Leonberg**, Kreiskrankenhaus **Leonberg**, H.P. Stegbauer; **Lörrach**, Kreiskrankenhaus Lörrach, H.F. Ginz; **Lübeck**, Universitätsklinikum Schleswig Holstein—Campus Lübeck, B. Sedemund-Adib; **Lüneburg**, Städtisches Klinikum Lüneburg, C. Frenkel, D. Yakisan, H. Schröder, C. Daniels; **Lünen**, St.-Marien-Hospital, M. Burrichter, T. Bernhardt, W. Wilhelm; **Lutherstadt Wittenberg**, Paul-Gerhard-Stiftung, Speck, P. Jehle; **Magdeburg**, Universitätsklinikum Magdeburg, W. Brandt; **Mannheim**, Universitätsklinikum Mannheim, T. Luecke, A. Gruener; **Mönchengladbach**, Krankenhaus Neuwerk, A. Keller, S. Scieszka; **Mühlhausen**, Unstrut-Hainich Kreiskrankenhaus Mühlhausen, S. Frenzel, L. Pfeiffer; **München**, Krankenhaus der Barmherzigen Brüder München, F. Brettner; **Münster**, Universitätsklinikum Münster, K. Eicker, F. Hinder; **Neubrandenburg**, Dietrich-Bonhoeffer-Klinikum-Neubrandenburg, M. Schneider; **Neuruppin**, Ruppiner Klinikum GmbH, T. Nippraschk, D. Hoffmeister; **Northeim**, Albert-Schweitzer-Krankenhaus Northeim, M. Bund; **Regensburg**, Klinikum der Universität Regensburg, H. Künzig, T. Bein; **Regensburg**, Krankenhaus der Barmherzigen Brüder Regensburg, A. Speicher; **Rochlitz**, Kreiskrankenhaus Rochlitz, P. Klut; **Rostock**, Medizinische Fakultät der Universität Rostock, D.A. Vagts, G. Nöldge-Schomburg; **Salzgitter**, Klinikum Salzgitter GmbH, J. Offensand, S. Youssef, J.-P. Juvana; **Schwerte**, Evang. Krankenhaus Schwerte GmbH, K. Schwarke; **Sindelfingen**, Ermstalklinik Städtisches Krankenhaus Sindelfingen, J. Fritschi, P. Zaar; **Speyer**, St.-Vincentius-Krankenhaus Speyer, K.P. Wresch, K. Steidel; **Stadtoldendorf**, Kreiskrankenhaus Charlottenstift, M. Hundt, U. Schulze, J. Kolle; **Stuttgart**, Robert-Bosch-Krankenhaus, G. Meinhardt; **Traunstein**, Klinikum Traunstein, M. Glaser, T.P. Zucker; **Trier**, Krankenhaus der Barmherzigen Brüder Trier, A. Deller; **Troisdorf**, St. Johannes-Krankenhaus Troisdorf, W. Theelen; **Ulm**, Bundeswehrkrankenhaus Ulm, M. Burkert; **Ulm**, Universitätsklinikum Ulm, E. Calzia; **Wangen**, Fachkliniken Wangen, Dr J. Jahn, A. Schneider; **Wolfenbüttel**, Städtisches Klinikum Wolfenbüttel, W. Seyde; **Würzburg**, Universitätsklinik Würzburg, J. Brederlau, E. Kaufmann, F. Schuster, C. Söllmann; **Zeitz**, Georgius-Agricola-Klinikum Zeitz, J. Haberkorn.

## Additional file

Additional file 1: Supplemental material with (i.) data comparing patients with and without ARDS, (ii.) multivariate analyses in non-ARDS patients and (iii.) detailed descriptions of the multivariable models. (DOC 294 kb)

## Abbreviations

ALI: Acute lung injury
ALIEN: Acute Lung Injury: Epidemiology and Natural History
ARDS: Acute respiratory distress syndrome
ARF: Acute respiratory failure
AUC: Area under the curve
BIPAP: Biphasic positive airway pressure
BMI: Body mass index
CI: Confidence interval
FiO_2_: Fraction of inspired oxygen
ICU: Intensive care unit
IQR: Interquartile range
KCLIP study: King County Lung Injury Project
NIV: Non-invasive ventilation
OR: Odds ratio
PaCO_2_: Partial pressure of arterial carbon dioxide tension
PaO_2_: Partial pressure of arterial oxygen tension
PaO_2_/FiO_2_: Arterial-to-inspired-oxygen
PEEP: Positive end-expiratory pressure
ROC: Receiver-operator characteristic
SAPS II: Simplified Acute Physiology Score II
SD: Standard deviation
VENTILA study: International Study of Mechanical Ventilation
