# Supplementary material for: Outcome of acute respiratory distress syndrome in university and non-university hospitals in Germany
Source: Crit Care. 2017 May 30;21:122. doi: 10.1186/s13054-017-1687-0 (PMC5448143; doi:10.1186/s13054-017-1687-0)
Supplement: Additional file 1: — Supplemental material with (i.) data comparing patients with and without ARDS, (ii.) multivariate analyses in non-ARDS patients and (iii.) detailed descriptions of the multivariable models. (DOC 294 kb) [file 13054_2017_1687_MOESM1_ESM.doc]

Additional file 1

**Outcome of**

**acute respiratory distress syndrome**

**in university and non-university hospitals**

**in Germany**

Konstantinos Raymondos, Michael Quintel, Tamme Dirks, Ulrich Molitoris, Jörg Ahrens,

Thorben Dieck, Kai Johanning, Dietrich Henzler, Rolf Rossaint, Christian Putensen,

Hermann Wrigge, Ralph Wittich, Maximilian Ragaller, Thomas Bein, Martin Beiderlinden, Maxi Sanmann, Christian Rabe, Jörn Schlechtweg, Monika Holler, Fernando Frutos-Vivar, Andres Esteban, Hartmut Hecker, Simone Rosseau, Vera von Dossow,

Claudia Spies, Tobias Welte, Siegfried Piepenbrock, Steffen Weber-Carstens

| **Table S1: Characteristics on ICU admission and outcome of patients with and without ARDS** | | | | |
| --- | --- | --- | --- | --- |
|  | **All patients** | **ARDS** | **Without ARDS** |  |
| **Mean (SD); median (IQR) or n (%)** | *(n=1028)* | *(n=198)* | *(n=830)* | *p-value* |
| Age, years | 61.5 (16.3); 65 (51–74) | 61.1 (16.1); 64 (50–73) | 61.6 (16.3); 65 (51–74) | *0.68* |
| Sex, male/female | 653 (63.8)/371 (36.2) | 142 (71.7)/56 (28.3) | 511 (61.9)/315 (38.1) | *0.01* |
| SAPS II score at ICU admission | 40.6 (18.9); 38 (27–52) | 47.0 (19.0); 46 (34–60) | 39.1 (18.6); 36 (26–50) | *<0.001* |
| Main problem, medical/surgical | 379 (36.9)/647 (63.1) | 117 (59.1)/81 (40.9) | 262 (31.6)/566 (68.4) | *<0.001* |
| BMI, kg/m² | 25.4 (23.0–28.3) | 25.8 (23.2–28.4) | 25.4 (23.0–28.2) | *0.22* |
| **Reason for the initiation of mechanical ventilation** | (n=1028) | (n=181)*** | (n=830) |  |
| **Acute respiratory failure** |  |  |  |  |
| ARDS | 17 (1.7) | - | - | *-* |
| Postoperative | 387 (37.6) | 31 (17.1) | 356 (42.9) | *<0.001* |
| Aspiration | 29 (2.8) | 14 (7.7) | 15 (1.8) | *<0.001* |
| Community acquired pneumonia | 57 (5.5) | 24 (13.3) | 33 (4.0) | *<0.001* |
| Hospital acquired pneumonia | 35 (3.4) | 15 (8.3) | 20 (2.4) | *<0.001* |
| Sepsis | 81 (7.9) | 22 (12.2) | 59 (7.1) | *0.03* |
| Trauma | 64 (6.2) | 13 (7.2) | 51 (6.1) | *0.61* |
| Congestive heart failure | 43 (4.2) | 10 (5.5) | 33 (4.0) | *0.41* |
| Cardiac arrest | 48 (4.7) | 9 (5.0) | 39 (4.7) | *0.85* |
| Other cause of acute respiratory failure | 66 (6.4) | 12 (6.6) | 54 (6.5) | *1* |
| **Acute respiratory failure due to chronic pulmonary disease** |  |  |  |  |
| COPD | 31 (3.0) | 9 (5.0) | 22 (2.7) | *0.15* |
| Asthma | 0 | 0 | 0 | *-* |
| Other chronic lung disease | 11 (1.1) | 2 (1.1) | 9 (1.1) | *1* |
| **Coma** |  |  |  |  |
| Metabolic coma | 15 (1.5) | 3 (1.7) | 12 (1.4) | *0.74* |
| Intoxication | 9 (0.9) | 2 (1.1) | 7 (0.8) | *0.67* |
| Hemorrhagic stroke | 52 (5.1) | 6 (3.3) | 46 (5.5) | *0.27* |
| Ischemic stroke | 20 (1.9) | 2 (1.1) | 18 (2.2) | *0.56* |
| Brain trauma | 50 (4.9) | 5 (2.8) | 45 (5.4) | *0.18* |
| Other coma | 1 (0.1) | 0 | 1 (0.1) | *1* |
| **Neuromuscular disease** | 11 (1.1) | 1 (0.6) | 10 (1.2) | *0.7* |
| Not specified | 1 (0.1) | 1 (0.6) | 0 | *0.18* |
| **Duration of mechanical ventilation, days** | 5 (2–11) | 12 (5–23) | 5 (2–10) | *<0.001* |
| **Length of ICU stay, days** | 9 (4–18) | 14 (8–29) | 8 (4–15) | *<0.001* |
| **Length of hospital stay, days** |  |  |  |  |
| All patients | 22 (13–37) | 26 (14–44) | 22 (13–36) | *0.07* |
| Survivors | 21 (13–35) | 31 (16–49) | 20 (12–33) | *<0.001* |
| **ICU mortality†** | 24.0 (242/1008) | 42.1 (82/195) | 19.7 (160/813) | *<0.001* |
| **Hospital mortality†** | 30.1 (298/989) | 49.5 (94/190) | 25.5 (204/799) | *<0.001* |
| SAPS II denotes severe acute physiology score II [1]; ICU, intensive care unit; BMI, Body Mass Index  *without ARDS as reason for the initiation of mechanical ventilation (n=17), †only for patients with known vital status at discharge | | | | |

| **Table S2: Overall prevalence of complications and organ failure in the course of mechanical ventilation** | | | | |
| --- | --- | --- | --- | --- |
|  | **Total** | **ARDS** | **Without ARDS** |  |
| n (%) | *(n=1028)* | *(n=198)* | *(n=830)* | *p-value* |
| Pneumonia | 255 (24.8) | 117 (59.1) | 138 (16.6) | *<0.001* |
| Sepsis | 286 (27.8) | 90 (45.5) | 196 (23.6) | *<0.001* |
| Renal failure | 240 (23.3) | 84 (42.4) | 156 (18.8) | *<0.001* |
| Liver failure | 57 (5.5) | 23 (11.6) | 34 (4.1) | *<0.001* |
| Cardiovascular failure | 614 (59.7) | 157 (79.3) | 457 (55.1) | *<0.001* |
| Coagulopathy | 123 (12.0) | 52 (26.3) | 71 (8.6) | *<0.001* |
| Respiratory acidosis | 176 (17.1) | 66 (33.3) | 110 (13.3) | *<0.001* |
| Metabolic acidosis | 112 (11.6) | 36 (18.2) | 76 (9.2) | *0.001* |
| Barotrauma | 56 (5.4) | 29 (14.6) | 27 (3.3) | *<0.001* |

| **Table S3:** **Univariable and multivariable analysis of factors associated with hospital mortality in ventilated patients without ARDS** | | | | |
| --- | --- | --- | --- | --- |
| **Variable** | **Univariable analysis*** | | **Multivariable analysis†** | |
|  | Odds ratio  (95% CI) | *p-value* | Odds ratio  (95% CI) | *p-value* |
| **Factors related to patients’ baseline factors** | | | | |
| **SAPS II score at ICU admission** |  | *0.038* |  | *<0.001* |
| ≤ 40 | 1 |  | 1 |  |
| 40–59 | 1.65 (0.91–3.0) |  | 2.30 (1.46–3.64) |  |
| ≥ 60 | 2.13 (1.16–3.9) |  | 3.54 (1.97–6.36) |  |
| **Reason for the initiation of mechanical ventilation** |  | *<0.001* |  | *<0.001* |
| Postoperative | 1 |  | 1 |  |
| Sepsis | 10.38 (5.57–19.33) |  | 4.21 (1.90–9.35) |  |
| Coma | 3.02 (1.89–4.81) |  | 2.90 (1.63–5.15) |  |
| Pulmonary etiology of respiratory failure without pneumonia | 2.10 (1.02–4.33) |  | 1.49 (0.60–3.70) |  |
| Other reasons for acute respiratory failure | 2.43 (1.56–3.78) |  | 1.15 (0.65–2.05) |  |
| Pneumonia | 1.27 (0.58–2.76) |  | 0.50 (0.20–1.27) |  |
| **Factors related to individual patient management** | | | | |
| **pHa** |  | *<0.001* |  | *0.003* |
| Acidosis (<7.35) | 4.21 (2.73–6.49) |  | 2.57 (1.45–4.54) |  |
| 7.35–7.45 | 1 |  | 1 |  |
| Alkalosis (≥7.46) | 1.16 (0.75–1.80) |  | 1.56 (0.91–2.67) |  |
| Respiratory frequency, breaths/min | 1.08 (1.04–1.12) | *<0.001* |  |  |
| FiO_2_ (0.1 increments) | 1.37 (1.18–1.60) | *<0.001* | 1.27 (1.04–1.55) | *0.02* |
| Use of vasoactive drugs on 2 consecutive days | 2.45 (1.76–3.41) | *<0.001* |  |  |
| **Factors developing during mechanical ventilation** | | | | |
| Renal failure | 6.31 (4.32–9.21) | *<0.001* | 3.43 (2.12–5.56) | *<0.001* |
| Coagulopathy | 4.76 (2.86–7.92) | *<0.001* | 2.76 (1.44–5.29) | *0.002* |
| Cardiovascular failure | 3.10 (2.17–4.41) | *<0.001* | 2.15 (1.38–3.35) | *0.001* |
| **pHa** |  | *<0.001* |  | *0.003* |
| Acidosis (<7.35) | 4.21 (2.73–6.49) |  | 2.57 (1.45–4.54) |  |
| 7.35–7.45 | 1 |  | 1 |  |
| Alkalosis (≥7.46) | 1.16 (0.75–1.80) |  | 1.56 (0.91–2.67) |  |
| Metabolic acidosis | 7.21 (4.31–12.04) | *<0.001* |  |  |
| **Factors related to the setting of care** | | | | |
| Number of ventilated patients / ICU (increments of 10 patients/month) | 0.78 (0.69–0.88) | *<0.001* | 0.82 (0.70–0.95) | *0.01* |
| **ICU size, number of ICU beds** |  | *0.001* |  |  |
| ≤8 | 1.18 (0.67–2.11) |  |  |  |
| 8–10 | 2.15 (1.45–3.19) |  |  |  |
| 11–13 | 0.96 (0.63–1.47) |  |  |  |
| ≥14 | 1 |  |  |  |
| **ICU specialization** |  | *<0.001* |  |  |
| Surgical | 1 |  |  |  |
| Medical | 1.53 (0.93–2.52) |  |  |  |
| Surgical/medical or neurological | 2.15 (1.52–3.04) |  |  |  |
| SAPS II denotes severe acute physiology score II^1^; ICU, intensive care unit; FiO_2_, fraction of inspired oxygen; driving pressure = plateau pressure – PEEP; PBW, predicted body weight; ARF, acute respiratory failure  *Only those variables are shown that qualified with a p-value < 0.1 in the four prior multivariate analyses within the four variable categories for the overall final multivariable model.  †Only those variables are shown that remained significant in the final multivariable model for n=737 with stepwise backward elimination using a threshold of p=0.05 (according to Wald statistics). Goodness of fit: Hosmer-Lemeshow-test [2]: p=0.269; area under the receiver-operator curve: 0.83 (95% CI 0.79–0.86), p<0.001 | | | | |

**Logistic regression variables and building of the multivariable models for patients with ARDS and for patients without ARDS**

In a similar manner to Esteban et al. in the First VENTILA study, we grouped all potential prognostic factors into three distinct categories [3], namely **a)** *patients’ baseline factors*, **b)** *factors related to individual patient management*, and **c)** *events occurring during the course of mechanical ventilation*. We also introduced a fourth category to our models, incorporating **d)** *factors related to the setting of care of the treating ICU*.

To allow different combinations of prognostic factors in the two different patient groups with respect to the development of ARDS, and also because some factors were applicable in only one of the two groups, multivariable analyses were performed separately for patients with and without ARDS.

Ventilator and gas exchange variables were coded as mean values from the first week after ARDS onset, or after initiation of mechanical ventilation in patients without ARDS. In the same manner as Ferguson et al., we chose this interval to reflect the acute phase of illness instead of collecting data from patients either with further complications or in their recovery phase [4]. Predicted body weight was calculated in accordance with the ARDS Network study [5].

As a first step, variables associated with hospital mortality were identified by univariable logistic regression, with all variables in both patient groups examined. A given variable was further considered for both models when associated with hospital mortality with a p-value < 0.15 in one or in both groups. This was done to avoid missing a possibly important factor due to the lower sample size of the patient group with ARDS, and to allow the same potential factors in both groups to qualify as risk factors, where applicable. Additionally, predefined factors of clinical importance were selected.

When univariable analysis produced a p-value > 0.15 for variables in both patient groups but yielded potential clinical relevance, they were further examined in both groups and included in the corresponding variable groups. These variables were **a)** body mass index (BMI), and (only within the ARDS group) timing of ARDS onset (late vs. early onset); **b)** unsuccessful use of non-invasive ventilation (NIV), prone positioning on at least one day within the first week after onset of ARDS in patients with ARDS, or after initiation of ventilation in patients without ARDS; **c)** sepsis and acute lung injury (only within the group of patients without ARDS) [6].

The following variables yielded a p-value > 0.15 in the univariable analysis for both patient groups, and were excluded from further analyses: **a)** weight, height, tracheostomy present at start of ventilation, initiation of ventilation prior to ICU admission, initiation of ventilation prior to hospital admission; **b)** use of invasive ventilation at start of ventilation; **d)** availability of NIV as standard procedure on the ICU. In both groups, use of neuromuscular blockers on two consecutive days was excluded because there were too few patients (< 10) per factor. There were also too few ‘positive patients’ with respect to the successful use of NIV in ARDS. Mean peak pressure was excluded because it was unavailable in 62% of patients and strongly correlated (r > 0.6) with plateau pressure.

Continuous variables exhibiting a non-linear relationship with mortality in our data, or in previous studies, were grouped according to previously published thresholds or quartiles: age (< 40, 40-69, ≥ 70 years) [4], SAPS II (< 40, 40-59, ≥ 60 points) [1,3], BMI (< 18.5, 18.5-24.99, 25.0-29.99, ≥ 30.0 kg/m²) for patients without ARDS; because only six patients with ARDS were underweight, the first category was coded < 25.0 kg/m² for patients with ARDS. For both ventilatory and gas exchange variables, we used the mean values from the first week after onset of ARDS in patients with ARDS, or the mean values from the first week after the initiation of ventilation in patients without ARDS: tidal volume/kg predicted body weight (PBW) (< 8.0, 8.0-9.99, ≥ 10.0 ml/kg PBW), PEEP (≤ 5, 6-10, ≥ 11 cmH_2_O), pH value (< 7.35, 7.35-7.45, ≥ 7.46)). The ICU size was grouped according to the quartiles of the 95 participating ICUs (≤ 7, 8-10, 11-13, ≥ 14 beds), and hospital size according to the quartiles of the 80 participating hospitals (≤ 315, 316-460, 461-900, ≥ 901 beds).

All selected variables were grouped into the four variable categories mentioned above:

**a) Patient’s baseline factors:** Age, sex, simplified acute physiology score (SAPS) II [1], main problem (medical vs. surgical), main reason for initiation of ventilation (pneumonia, lung disease other than pneumonia, sepsis, postoperative status, other acute respiratory failure, neurological reason), length of hospital stay prior to ventilation, and (only within the ARDS group): type of lung injury (extrapulmonary vs. pulmonary);

**b) Factors related to individual patient management:** Tidal volume per kg PBW, respiratory rate, plateau pressure, applied PEEP, FiO_2_, PaO_2_/FiO_2_ ratio, pH, respiratory system compliance and driving pressure (mean of all ventilatory and gas exchange variables from the first week after onset of ARDS in patients with ARDS, or from the first week after initiation of ventilation in patients without ARDS), successful use of NIV, high plateau pressure (>30 cmH_2_O) on two consecutive days, and use of medications on two consecutive days (sedatives, vasoactive drugs);

**c) Events occurring during the course of mechanical ventilation:** Occurrence of metabolic acidosis (pH < 7.3 and PaCO_2_ < 45 mmHg), respiratory acidosis (pH < 7.35 and PaCO_2_ > 55 mmHg), pneumonia, barotrauma, hepatic failure, renal failure, shock, coagulopathy, lowest PaO_2_/FiO_2_ ratio;

**d) Factors related to the setting of care:** university/non-university hospital, size of hospital, size of ICU, type of ICU, ICU volume of all admitted patients, ICU volume of mechanically ventilated patients, and ICU volume of mechanically ventilated patients per bed.

Using multivariable logistic regression, the selected variables were then analyzed separately for the group of patients with ARDS and without ARDS. Entering too many predictors into a model and collinearity between predictors can lead to unstable coefficient estimation. Because numerous variables needed to be taken into account, the approach adopted to correct for these issues was as follows: a stepwise backward selection procedure was used to perform separate, multivariable ranking of the factors within each of the four variable categories. As a result, only those variables contributing with a p-value < 0.10 to the multivariable analysis per variable category were entered into the two overall final models, thereby correcting for collinearity of predictors. We chose this p-value to reduce the number of predictors without excluding potentially important factors close to statistical significance.

In the group of patients *with* ARDS, the variables that contributed with a p-value < 0.10 and subsequently entered into the final model for multivariable logistic regression were: **a)** sex, simplified acute physiology score II (SAPS II); **b)** ventilator settings and gas exchange variables (mean value) from the first week after onset of ARDS (FiO_2_, driving pressure, pH value); use of vasoactive drugs on two consecutive days, **c)** complications and organ dysfunctions (metabolic acidosis, hepatic failure, renal failure, lowest PaO_2_/FiO_2_ ratio) and **d)** university/non-university hospital.

In the group of patients *without* ARDS, the variables contributing with a p-value < 0.10 and subsequently entered into the final model were: **a)** SAPS II, length of hospital stay prior to ventilation, main reason for initiation of ventilation (pneumonia, lung disease other than pneumonia, sepsis, postoperative status, other acute respiratory failure, neurological reason); **b)** ventilator settings and gas exchange variables (mean value) from the first week after the onset of ventilation (FiO_2_, respiratory rate, pH value); use of vasoactive drugs on two consecutive days, **c)** complications and organ dysfunctions (metabolic acidosis, renal failure, shock, coagulopathy) and **d)** ICU volume of ventilated patients, size of ICU and type of ICU.

The final model for the group of patients with ARDS, and the final model for the group of patients without ARDS, were built using a stepwise backward elimination procedure, excluding variables from the model showing no significant contribution (threshold p=0.05). All p-values in the logistic regression were reported according to Wald statistics.

The goodness of fit of the final models was assessed using the Hosmer-Lemeshow test, and a p-value of > 0.1 indicated good agreement between observed and predicted mortality [2]. Discrimination ability was analyzed with reference to the receiver-operator characteristic (ROC), and its area under the curve was calculated for the two final models for patients with and without ARDS.


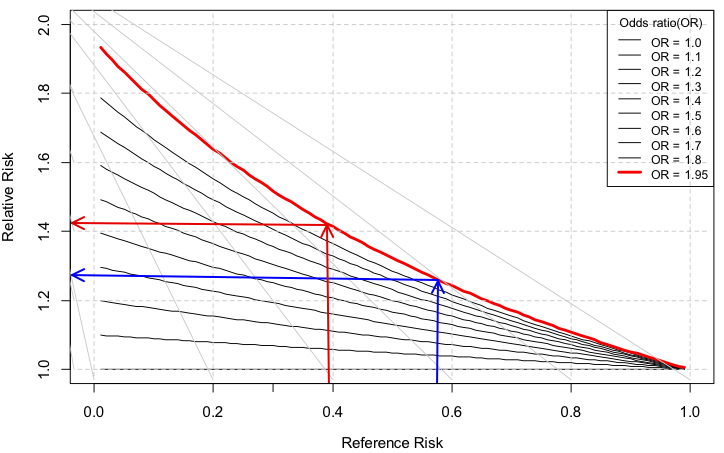


**Figure S1. Relationship between relative risk and odds ratio (OR) in relation to the reference risk.** The OR of 1.1 for a 1 cmH_2_O increment of driving pressure found in the present study corresponds to an OR of 1.95 (=1.1^7^) for a 7 cmH_2_O increment of driving pressure (red line). This increment of driving pressure was reported by Amato et al. to be associated with an increased mortality as indicated by a relative risk of 1.41 (95% confidence interval (CI), 1.31-1.51) [7]. According to the reference risk for ARDS patients in university hospitals in the present study (hospital mortality 39.3%, (CI 28.6-49.9%) this OR of 1.95 corresponds to a very similar relative risk (red arrows) when compared to the relative risk reported by Amato et al. In contrast, according to the higher reference risk for ARDS patients in non-university hospitals (hospital mortality 57.5%, (CI 48.0-67.1%) this OR of 1.95 corresponds to a lower relative risk in non-university hospitals (blue arrows).

**References**

1. Le Gall JR1, Lemeshow S, Saulnier F. A new Simplified Acute Physiology Score (SAPS II) based on a European/North American multicenter study. *JAMA* 1993; 270(24): 2957-63.

2. Hosmer DW, Lemeshow S: Applied Logistic Regression. New York, John Wiley and Sons, 1989.

3. Esteban A, Anzueto A, Frutos F, Alía I, Brochard L, Stewart TE, Benito S, Epstein SK, Apezteguía C, Nightingale P, Arroliga AC, Tobin MJ; Mechanical Ventilation International Study Group. Characteristics and outcomes in adult patients receiving mechanical ventilation: a 28-day international study. *JAMA* 2002; 287(3): 345-55.

4. Ferguson ND, Frutos-Vivar F, Esteban A, Anzueto A, Alía I, Brower RG, Stewart TE, Apezteguía C, González M, Soto L, Abroug F, Brochard L; Mechanical Ventilation International Study Group. Airway pressures, tidal volumes, and mortality in patients with acute respiratory distress syndrome. *Crit Care Med* 2005; 33(1): 21-30.

5. Brower RG, Matthay MA, Morris A, Schoenfeld D, Thompson BT, Wheeler A. Ventilation with lower tidal volumes as compared with traditional tidal volumes for acute lung injury and the acute respiratory distress syndrome. *N Engl J Med* 2000; 342(18): 1301-8.

6. Bernard GR, Artigas A, Brigham KL, Carlet J, Falke K, Hudson L, Lamy M, Legall JR, Morris A, Spragg R. The American-European Consensus Conference on ARDS. Definitions, mechanisms, relevant outcomes, and clinical trial coordination. *Am J Respir* *Crit Care Med* 1994; 149(3 Pt 1): 818-24.

7. Amato MB, Meade MO, Slutsky AS, Brochard L, Costa EL, Schoenfeld DA, Stewart TE, Briel M, Talmor D, Mercat A, Richard JC, Carvalho CR, Brower RG. Driving pressure and survival in the acute respiratory distress syndrome. *N Engl J Med* 2015; 372:747-755.
